# N-Accesses: A Blockchain-Based Access Control Framework for Secure IoT Data Management

**DOI:** 10.3390/s23208535

**Published:** 2023-10-18

**Authors:** Teng Hu, Siqi Yang, Yanping Wang, Gongliang Li, Yulong Wang, Gang Wang, Mingyong Yin

**Affiliations:** 1 Institute of Computer Application, China Academy of Engineering Physics, Mianyang 621900, China; mailhuteng@gmail.com (T.H.); siqirdj@hotmail.com (S.Y.); ligongliang1982@126.com (G.L.); wangyulong@caep.cn (Y.W.); gary.wang@caep.cn (G.W.); 2 School of Computer Science and Engineering, University of Electronic Science and Technology of China, Chengdu 611731, China; wangyp1108@gmail.com

**Keywords:** blockchain, smart contract, access control, data management

## Abstract

With the rapid advancement of network communication and big data technologies, the Internet of Things (IoT) has permeated every facet of our lives. Meanwhile, the interconnected IoT devices have generated a substantial volume of data, which possess both economic and strategic value. However, owing to the inherently open nature of IoT environments and the limited capabilities and the distributed deployment of IoT devices, traditional access control methods fall short in addressing the challenges of secure IoT data management. On the one hand, the single point of failure issue is inevitable for the centralized access control schemes. On the other hand, most decentralized access control schemes still face problems such as token underutilization, the insecure distribution of user permissions, and inefficiency.This paper introduces a blockchain-based access control framework to address these challenges. Specifically, the proposed framework enables data owners to host their data and achieves user-defined lightweight data management. Additionally, through the strategic amalgamation of smart contracts and hash-chains, our access control scheme can limit the number of times (i.e., n-times access) a user can access the IoT data before the deadline. This also means that users can utilize their tokens multiple times (predefined by the data owner) within the deadline, thereby improving token utilization while ensuring strict access control. Furthermore, by leveraging the intrinsic characteristics of blockchain, our framework allows data owners to gain capabilities for auditing the access records of their data and verifying them. To empirically validate the effectiveness of our proposed framework and approach, we conducted extensive simulations, and the experimental results demonstrated the feasibility and efficiency of our solution.

## 1. Introduction

The rapid evolution of communication and networking technologies has spurred the proliferation of sensors connected to the Internet, giving rise to the concept of the Internet of Things (IoT). IoT devices have found widespread adoption across various domains, including autonomous driving systems, smart homes, robotics, and intelligent healthcare systems. This pervasive adoption has led to the generation and collection of vast volumes of IoT data, which frequently traverse the boundary between the physical world and computational components.

In the data-driven era, data have emerged as a new form of currency, holding immeasurable value. The private data on specific IoT devices can be combined with intelligent platforms to provide assistance for future research and predictions, which is especially valuable. Thus, enhancing secure IoT data management is an imperative task. Building upon the research efforts of [1,2], secure data management includes three key components:

1. Confidentiality: Data should be provided encryption and hashing to ensure data security.
2. Access control: Different access rights for different users should be set, and the accesses should be delegated by the data owner.
3. License management: The license (keys, authentication code, etc.) to authorized users should be trust-released and efficiently managed. Furthermore, it is necessary to control and check the access condition (the number of access times, the lifetime, etc.).

However, in today’s complex network environments, IoT data are susceptible to security and privacy attacks, making secure data management especially challenging. Specifically, in many application scenarios, data are often transmitted and stored in an unprotected state, leading to security threats such as eavesdropping, theft, and tampering attacks. Besides, most current IoT-data-management frameworks [3] rely on cloud servers to provide third-party services for managing privacy-sensitive data. However, this centralized data management may entail significant security risks (e.g., a single point of failure) and also result in data owners losing control of their data.

The decentralized infrastructure of blockchain technology has the potential to fortify the security and credibility of the management of IoT data. Unlike traditional centralized architectures, blockchain does not rely on specific central nodes for data processing and storage, so that it can enhance system robustness and mitigate centralized management problems. Furthermore, it offers the properties of accountability, trust, and non-repudiation, making it a promising technology for expanding data sharing while addressing trust-related challenges [4,5].

Accordingly, a multitude of researchers have diligently devoted their efforts to the domains of blockchain-based IoT data management. In particular, Ouaddah et al. [6] introduced a privacy-preserving blockchain-based IoT access-control framework, wherein token operations (grant, acquisition, delegation, and revocation) are implemented as blockchain transactions. Nevertheless, this scheme [6] restricts one token from accessing only one resource, resulting in tokens’ underutilization and also posing challenges in token management when resource volumes increase. Lyu et al. [7] presented a blockchain-based access-control framework, affording data owners the capacity to securely distribute, audit, and revoke access rights. However, as observed in [8], the distribution of user permissions occurs externally to the blockchain, rendering user rights susceptible to tampering. To achieve fine-grained secure access control, Wang et al. [9] proposed a blockchain-based data-sharing scheme, where data owners encrypt their shared data using attribute-based encryption. However, the scheme [9] entails high execution costs due to the involvement of intricate cryptographic operations, making it unsuitable for moderately complex blockchain scenarios.

In light of the above analysis, we identified prevailing issues in current blockchain-based access-control frameworks, including token underutilization, insecure user permission distribution, and inefficiency. To address these challenges, this paper introduces a lightweight blockchain-based access-control framework tailored to secure IoT data management. Our framework empowers data owners to directly host their IoT data, granting them the autonomy to efficiently determine who can access their data, the frequency of access, and the access time frame. Besides, by leveraging the inherent characteristics of blockchain, data owners can conduct audits to ascertain who has accessed their data and verify access records, which enhances transparency and trust in data management systems.

   **Contributions:** The primary contributions of this paper are as follows:

1. *A blockchain-based access control framework with configurable restrictions is proposed.* Our framework enables data owners to host their data, achieving the goal of lightweight data access control. In addition, it facilitates trusted audits and ensures non-repudiation of permissions.
2. *The n-times access control approach based on hash chains and smart contracts was achieved.* We propose an innovative access-control mechanism where authorized users have a limited number of access instances before a specified deadline. This approach optimizes token utilization while preserving robust security. Furthermore, the entire process is automatically executed through smart contracts, and user access behavior is immutably recorded on the blockchain.
3. *Empirical verification of feasibility and efficiency is conducted.* To validate the practicality and efficiency of our framework and approach, we conducted comprehensive experiments, providing tangible evidence of their feasibility and efficiency in real-world scenarios.

## 2. Related Works

### 2.1. Access Control

Traditional access control can be roughly divided into three categories [10]: Discretionary Access Control (DAC), Mandatory Access Control (MAC), and Role-Based Access Control (RBAC) models.

The DAC model [11] allows the owner to authorize each access type (e.g., read, write, or execute) to a user based on the user’s identity or the groups the user belongs to. It can be achieved by several measures, such as Access Control Lists (ACLs), access matrices, and authorization tables. For example, Bui et al. [12] constructed a virtual software-defined LAN for users who want to exchange information, and the access control performed at the network level relies on physical addresses. The DAC schemes [12,13] are flexible. Nevertheless, they are not sufficiently restricted to enforce information flow policies: information passing from one user to another is unrestricted. Moreover, they are not scalable or efficient enough since the potential interconnection with devices of all kinds makes it burdensome to hold the complete data structure that this model proposes [10].

The Mandatory Access Control (MAC) model [14] defines a central authority that is responsible for deciding whether a user can access given information, and the user’s access is in a hierarchical way. Fan et al. [15] proposed a mandatory access control scheme to maintain the different levels of security for different types of objects (resource or data). Kumar and Tripathi [16] proposed a blockchain-based scalable access control scheme for the healthcare system. However, these schemes rely on the central structure, which is the main drawback since there is a central authority that determines what information can be accessed by which user, so transparency is not achieved.

The Role-Based Access Control (RBAC) model [17] was originally designed to simplify access management, which is under the principle that “a user’s responsibility is more important than the identity of the user”. In [18], an access control framework consisting of a Cyber–Physical Access Control Model (CPAC) and a Generalized Action Generation Model (GAGM) was proposed, which aims to augment the enforcement of authorization policies for healthcare. However, the scalability of the RBAC schemes [18,19,20] may be affected by the large number of dynamic users and roles that are involved in conglomerate networks [10], and moreover, the permission–role assignment is hard to change.

In summary, these traditional access-control models no longer meet the requirements of modern digital resources [21]. Thus, novel access-control frameworks with scalability and decentralizationshould be investigated.

### 2.2. Blockchain-Based Access Control for Data Management

Validating the access rights of subjects is usually conducted by a centralized entity in the traditional data-sharing scheme [3], which may cause a single point of failure. Due to its unique properties, the blockchain does not rely on specific central nodes to process and store data. Meanwhile, the blockchain can enable users to know where their data are stored and what is happening to their data.

Accordingly, the blockchain has been widely adopted in data management scenarios. In particular, Liang et al. introduced a data provenance platform, named ProvChain [22]; by leveraging the immutability and transparency features of blockchain, all data operation histories can be transparently and permanently recorded into the distributed ledger. Then, Maesa et al. [23] introduced a data-management mechanism, where the data consumer interacts with the data owner to negotiate a smart contract for automatically managing data. The smart contract in [23] is responsible for registering the sensor, requesting data, and providing financial functions. Gao et al. [5] proposed an IoT-data-sharing framework, in which the blockchain is used to authorize all devices in the network to improve their credibility and authenticity. However, data management in these works [5,23] requires the data owner to assign the decryption key; thus, the data owner is always online. To solve this problem, Truong et al. [24] proposed an IoT-data-sharing scheme that mitigated the online pressure of data owners, wherein the metadata are stored on-chain and the smart contract is used to control the access of users. However, the scheme of [24] requires a trusted party to assign the key to users, which makes the data management not very decentralized.

In addition, in some scenarios where it is necessary to protect user privacy and establish multi-party trust relationships, such as healthcare records, blockchain technology has been well applied. Xia et al. [25] proposed MeDShare based on blockchain, which addresses the problem of medical data sharing among medical big data custodians in a distrustful environment. Liu et al. [26] proposed a safe and effective way to achieve EMR data sharing, called BPDS, where the original EMR is stored securely in the cloud and the index is kept in the tamper-proof alliance blockchain. Kumar et al. [27] designed a novel secure and efficient data-sharing framework named PBDL, to solve the security and privacy issues with continuous communication over public networks. In their follow-up work, they further proposed optimization solutions [28]. They designed a blockchain-orchestrated deep learning approach named BDSDT. This approach can secure data transmission in IoT-enabled healthcare systems.

To achieve user-defined secure access control, Attribute-Based Encryption (ABE) is used in some blockchain-based systems. In particular, Wang et al. [9] proposed a data-sharing scheme based on blockchain, in which the data owner is responsible for distributing secret keys for data users and encrypting shared data using the ABE scheme to achieve fine-grained access control over the data. Buccafurri et al. [29] presented a scheme to combine smart contracts and blockchain with the ABE scheme. Although this scheme achieved access control, as well as service delivery with accountability requirements, it cannot be featured in some of the systems with computational restrictions.

Table 1 provides a comprehensive analysis of the current access-control models, from which we can find that traditional access-control models, such as DAC, MAC, and RBAC, have limitations in meeting the evolving demands of decentralized, transparent, and scalable data access control. Blockchain-based access control, leveraging blockchain’s unique properties, offers promising solutions, but still encounters challenges related to key management and computational restrictions. Thus, further investigations are needed to develop access control frameworks that effectively address the complex requirements of IoT data management.

## 3. Overview

In this section, we outline our framework and its goals.

### 3.1. Framework

**Figure 1:**
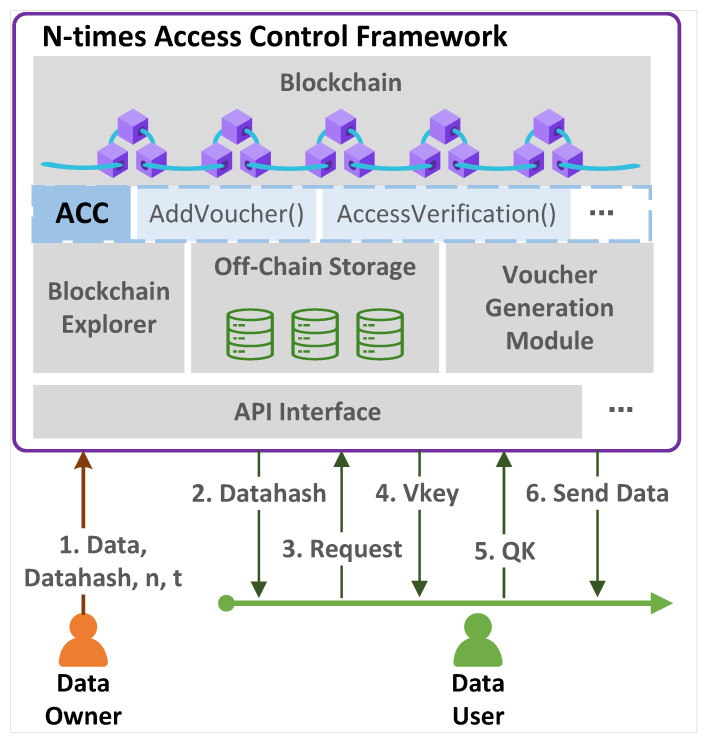
n-times access control framework.

As shown in Figure 1, our framework mainly consists of the blockchain, access control contract, blockchain explorer, off-chain storage, voucher-generation module, API, etc.

**Blockchain:** This is a distributed public ledger, where each network node maintains a replica of the ledger. In the framework, based on the blockchain, the smart contract is deployed to conduct the access control for users.

**ACC:** The Access Control Contract (ACC) mainly includes two functions: AddVoucher() and AccessVerification(). The former function is responsible for adding the voucher to the authorized list in the contract, while the latter is responsible for validating the QK sent by the user and returning the validation result to the API module.

**Off-chain storage module:** The DO’s data are off-chain stored, while the data’s meta-information (such as storage location, hash value, etc.) is stored on the blockchain.

**Blockchain explorer:** It provides an explorer interface for Users. The data user retrieves the data through the blockchain explorer and obtains the hash of the corresponding data.

**Voucher-generation module:** This is the core module for generating the voucher (*V*) and the DU’s authorization key ($V_{key}$). After receiving the call from the API, the module generates two random numbers ($x_{0}$, $x_{1}$) and calculates $V=(x_{n},x_{n+1},t)$ and $V_{key}=\left(x_{0}^{\prime },x_{1}^{\prime },n,t\right)$ based on the Datahash,n,t sent by the DO; the calculation approach is shown in Algorithm 1.

**API:** This is the interface between the user and the system. The interaction is completed through the API, and various functions of the framework are also triggered through API calls.

**Data Owner (DO) and Data User (DU):** The DO is any user/entity that owns and maintains some data, while the DU is anyone who wants to use the data. The DO delegates access rights (presets n,t), the data themselves (storage in off-chain storage), and the data hash (storage in the blockchain) to the framework. The DU obtains the data hash via the blockchain explorer and sends a request message to the API for the access key. Once the DU is authorized, the voucher-generation module computes *V* and $V_{key}$, then returns them to the ACC and DU, respectively. Therefore, the DU can use $V_{key}$ to access the data *n*-times before the deadline *t*.
**Algorithm 1** Generate HashChain**Input:** Random number: $x_{0},x_{1}$; data: *D*; number of access instances: *n* **Output:** HashChain  1:  Compute H(*D*), where H is a secure hash function.  2:  Compute HashChain[0]= H(H(*D*)$+x_{0}$) and HashChain[1]= H(H(*D*)$+x_{1}$)  3:  **for**
*i* in range (2,n+2,1) **do**  4:     HashChain[i]= H(HashChain[i−2]+HashChain[i−1])  5:  **end for**  6:  Finally, get: HashChain= [$hash_{1}$, $hash_{2}$, $hash_{3}$, …, $hash_{n}$, $hash_{n+1}$, $hash_{n+2}$]  7:  **return**
HashChain

### 3.2. Goals

The proposed framework achieves the following goals:

- **n-times authorized access control:** This means that only authorized users are allowed to access their required data. Additionally, instead of an infinite number of accesses, the user only has a limited number of access times. When the user uses up his/her chances, he/she will no longer have access to the data.
- **Trusted audit:** Benefiting from the properties of blockchain and smart contracts, each access request of the DU, the use of $V_{key}$, and other historical records can be verified by blockchain backtracking. Therefore, the trusted audit is ensured.
- **Offline one-to-many data access control:** The DO does not need to be online all the time. After delegating the data to the framework, the framework can complete the automatic authorization for different DUs, access right verification, etc., that is the realization of offline one-to-many data access control.

## 4. Approach

This section describes the concrete implementation of the proposed hash chain-based n-times access control approach. We first describe the process of the n-times access control and then introduce the smart contract we designed.

### 4.1. n-times Access Control Approach

We realized the control of the user’s access to the data through a hash chain and a smart contract. Expressly, our approach can limit the total number of times a user can access the data in a specific time range. For example, assuming that the authorized number of access times to a user is *n*, then the user can access the data at most *n*-times before the deadline. The n-times access control approach based on hash chain consists of three phases: voucher-generation phase, user-access phase, and permission-verification phase.

**Figure 2:**
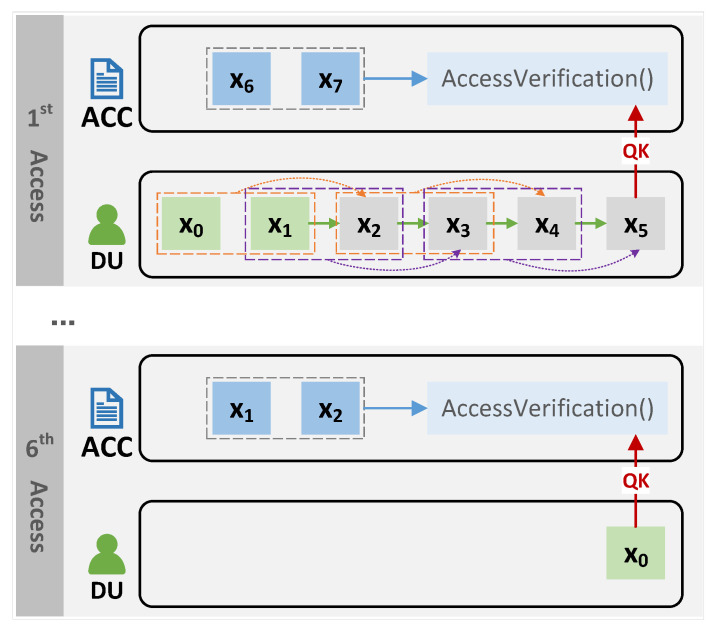
n-times access control approach.

Figure 2 shows an illustration of how our n-times access-control mechanism performs. In the illustration, the access times *n* was set to six. The DU has $V_{key}$, i.e., $\left(x_{0},x_{1},6,t\right)$, and $V=(x_{6},x_{7},t)$ is already stored in the ACC smart contract, where *t* is the deadline. Upon the first access, the DU sends $QK=x_{5}$, the ACC will use QK and *V* to determine whether the DU has access rights (the specific approach as described in Section 4.1.3), and if the DU passes the verification, the ACC will update *V*. The next time, the DU needs to pass the verification by sending $QK=x_{4}$; that is, $x_{5}$ can only be used once. In this way, the DU can pass the verification a total of six times. The last time, the DU sends $x_{0}$ to the smart contract to pass the verification. Since the DU cannot generate another QK to satisfy the verification condition in Section 4.1.3, the DU can no longer pass the verification.

#### 4.1.1. Voucher-Generation Phase

This phase is triggered by the DU, which retrieves the data’s meta-information in the blockchain and sends an access request to the framework. The detailed steps are as follows.

First of all, the DO sends the data, meta-information(include the data hash), *n*, and *t* to the framework via the API in advance. The framework stores the data in off-chain storage and the meta-information, *n*, and *t* in the blockchain.

1. The DU sends a request $Req=\{TAG_{i},ID_{d}\}$ to the framework for accessing, where $TAG_{i}$ is the unique identifier of the data that are included in the meta-information(e.g., the data hash), and $ID_{d}$ is the identity of the DU.
2. The framework will finalize the value of the access limit *n* (n∈Z) and the access time limit *t*, based on predefined values by the the DO or by asking the DO.
3. The framework first chooses two random numbers $x_{0},x_{1}$ and, then, generates a hash chain by Algorithm 1.
4. The framework sends $V_{key}=$ ($x_{0},x_{1},n,t$) to the user as a voucher for requesting access (via the API). After receiving the voucher $V_{key}$, the user needs to keep it properly (equivalent to the private key) and record how many times he/she has accessed the data, e.g., ($V_{key},i$), i.e., ($x_{0},x_{1},n,t,i$), where *i* means that *i*-times have been accessed.
5. The framework sends V= ($x_{n},x_{n+1},t$) to the Access Control Contract (ACC) as an authorized voucher. The ACC then stores the received certificate in the certificate table $V=[V_{1},V_{2},\ldots ,V_{i}]$ (i∈Z), which stores the access vouchers of different users in the form $V_{i}=$ ($v_{1},v_{2},t$) and used to verify the access rights.

#### 4.1.2. User Access Phase

When the user needs to access the data, the user first confirms that he/she has the authorization key for the data, then calculates the request key (QK) used for access and initiates an access request to the framework as follows:

1. According to the $V_{key}$ ($x_{0},x_{1},n,t,i$) for the data, the DU confirms that he/she has access to the data before *t*, and the remaining number of times is n−i.
2. The DU calculates the access request key (Query Key (QK)) according to *i*, then obtains $QK=x_{n-i}$. The calculation approach is the same as above and uses Algorithm 1.
3. The DU invokes the framework’s API and sends QK to request data access permission validation.

#### 4.1.3. Permission-Verification Phase

This phase enables a limited number of DU accesses to the data within the deadline. When an access permission verification request (i.e., QK) is received, the process is carried out as follows:

1. The ACC receives QK and starts traversing the vouchers table.
2. The ACC checks whether there exists ($v_{1},v_{2},t$) satisfying $v_{2}=H\left(QK+v_{1}\right)$, where H is a secure hash function.
3. The ACC checks whether the current time $t^{\prime }$ satisfies $t^{\prime }\leq t$.
4. If both Step 2 and Step 3 are satisfied, then the access verification is passed. The ACC replaces $v_{2}$ with $v_{1}$ and $v_{1}$ with QK in the voucher, i.e., the new voucher is updated to ($QK,v_{1},t$). The output fails otherwise.

### 4.2. Access Control Contract

We designed the smart contract for this system, the Access Control Contract (ACC). The ACC is responsible for verifying the access information and returning “Pass” or “Failed”. It contains two main functions:

- *AddVoucher*(): Once the framework has received the request from the DU, it will invoke this function to add the *V* of the DU to the ACC.
- *AccessVerification*(): Once the framework has received QK from the DU, it will invoke this function to verify if the DU has the access permission right to the data. This function will return 0 or 1 to the framework.

### 4.3. Analysis

Our proposed framework and approach achieved the properties of authorized access and limited access control for data security, as well as solved the problem of trusted auditing of data at the same time:

- **Authorized access:** Since the proposed framework ensures only the authorized user can obtain the access key $\left(x_{0},x_{1}\right)$, thereby only the authorized users can access the data. Meanwhile, smart contracts are exploited to control users’ access in the framework; anyone can verify whether specific access is valid, which also avoids the problem of untrustworthy authentication caused by traditional central servers.
- **Limited access:** In our access-control framework with configurable restrictions, once the user is authorized, he/she can obtain an n-times access key $V_{key}=\left(x_{0},x_{1},n,t\right)$, and the corresponding $V=(x_{n},x_{n+1},t)$ is added to the ACC. Due to the one-way nature of the hash function, the unauthorized DU without the correct key cannot pass the authentication operated by the smart contract. In addition, the length of the hash chain allocated to the user is limited to *n*. After the DU has used up his/her *n* times, the DU can no longer bypass the authentication, namely the DU can no longer access these data. Therefore, the proposed framework can achieve n-times limited access.

## 5. Experimental Simulation and Results’ Analysis

### 5.1. Simulation 1: Verify n-Times Access Control Functionality

**Figure 3:**
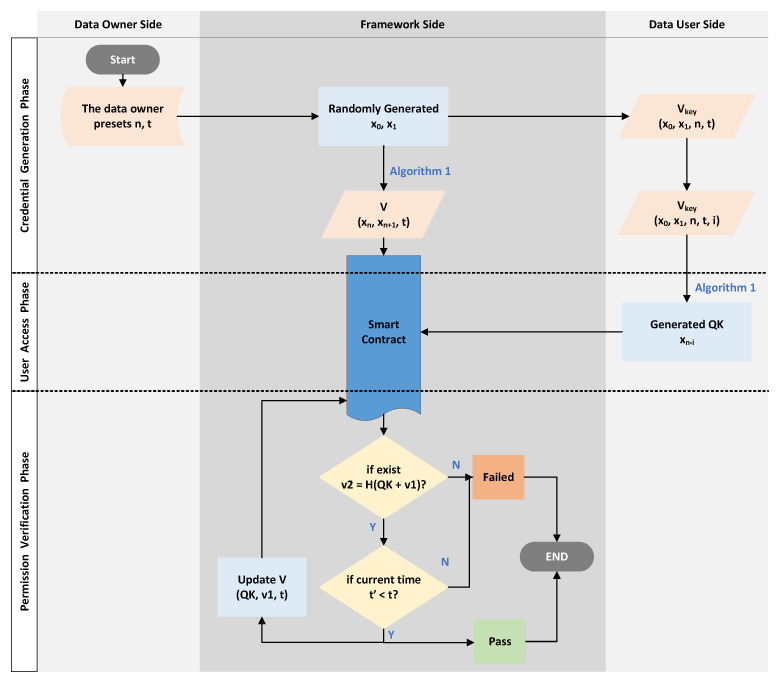
The illustration of Experimental Simulation 1.

This simulation was to verify the functionality of the framework mentioned in Section 3.1, the correctness of Algorithm 1, and the three phases of the n-times access control approach mentioned in this paper in Section 4.1. We designed the experimental simulation according to the process of Figure 3, as follows:

1. First, assume that the DO has stored the data in the off-chain storage and has stored the meta-information (including the data hash) into the blockchain. Set the default number of access times for the data to 8 (i.e., n=8), and the time allowed for access was restricted to 1 November 2022 (i.e., *t* = 2022-11-01 23:59:59);
2. When the framework receives an access request to the data, it invokes voucher-generation module to generate two random numbers $x_{0}$ and $x_{1}$ (the experiment used the random.getrandbits() function to generate a 128 bit-long random integer). Then, the voucher-generation module uses Algorithm 1 to calculate $x_{n}$ and $x_{n+1}$ and sends *V* and $V_{key}$ to the ACC and the requesting user (i.e., the DU), respectively.
3. The DU uses $V_{key}$ and Algorithm 1 to calculate QK and sends it to the framework as a request message via the API. In order to verify whether the DU can successfully pass the data access permission verification and when the number of access times exceeds *n* or the access time exceeds *t*, the permission verification cannot be passed.

**Figure 4:**
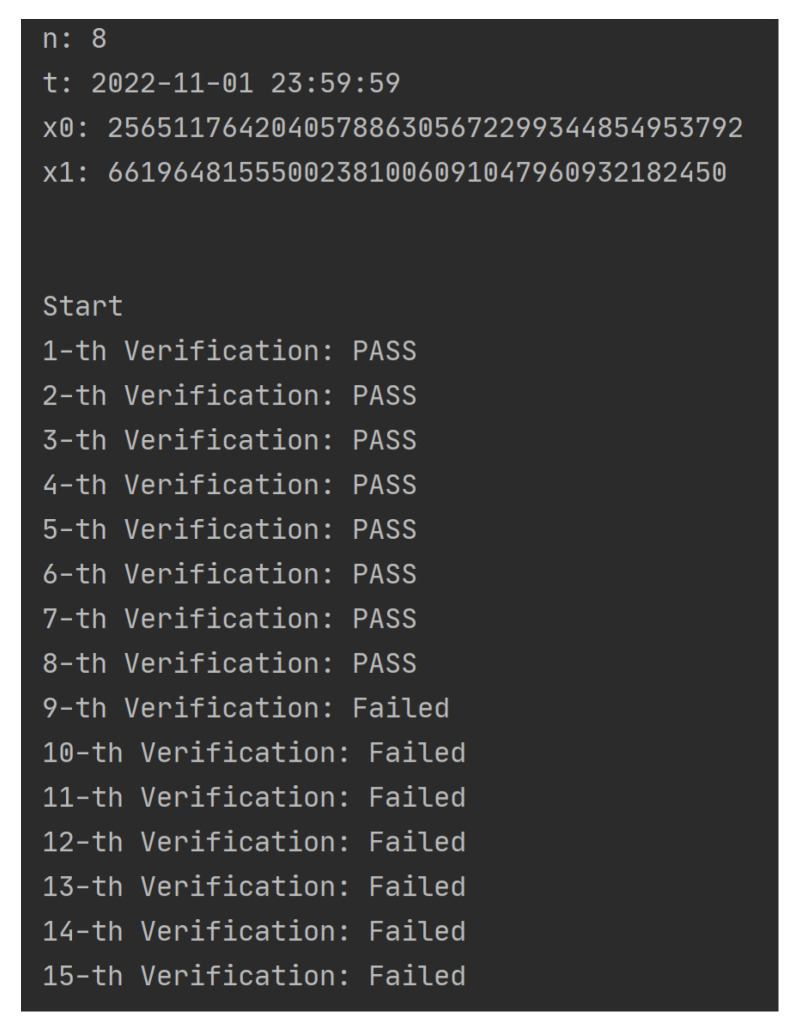
Simulation 1 results (Part A).

**Figure 5:**
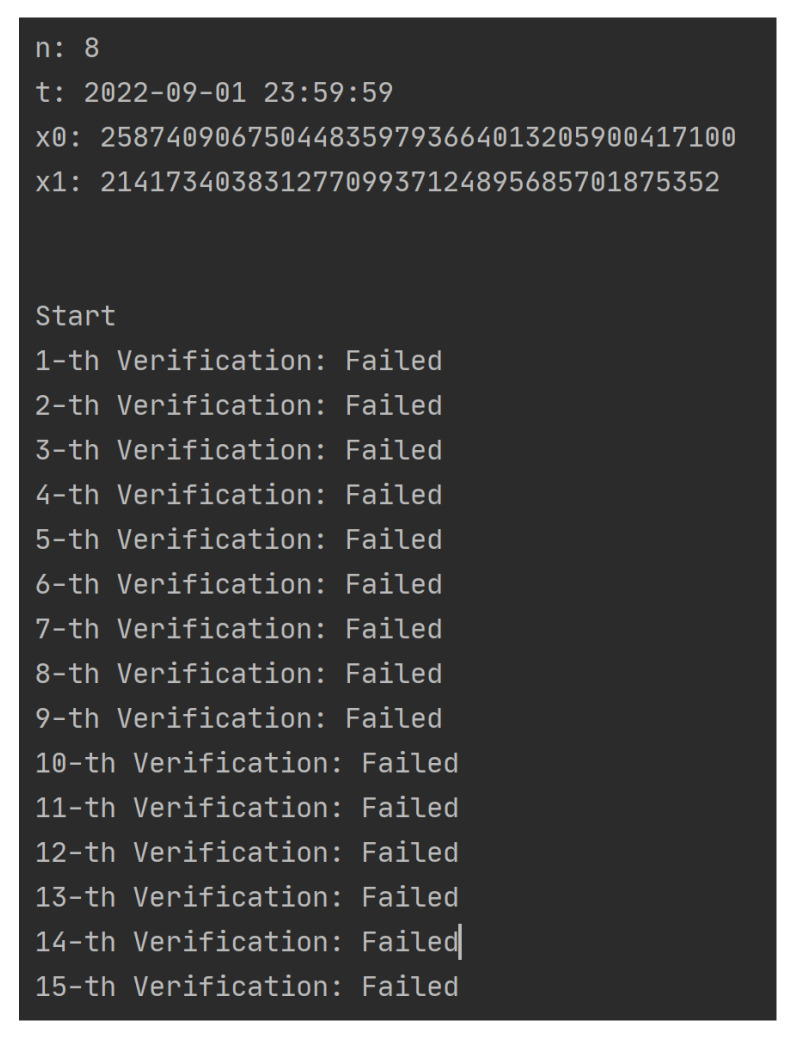
Simulation 1 results (Part B).

The experimental results were as follows:

1. When i≤n and $t^{\prime }\leq t$:
  As shown in Figure 4, *n* and *t* are preset values and $x_{0}$ and $x_{1}$ are randomly generated integers of length 128 bit; when the DU obtains $V_{key}$, he/she gains access to the data *n*-times before *t*; when the number of accesses *i* exceeds *n*, he/she will not pass the access verification. The specific process and results of each verification are shown in Table 2; the first eight times have passed the verification successfully, and each verification $v_{2}$ has been replaced by $v_{1}$ and $v_{1}$ replaced by QK. After more than eight times, no matter how much the DU tries (randomly generates some QK), he/she cannot pass the permission verification.
2. When i≤n and $t^{\prime }>t$:
  As shown in Figure 5, *n* and *t* are preset values and $x_{0}$ and $x_{1}$ are randomly generated integers of length 128 bit; when the DU obtains $V_{key}$, he/she obtains the permission to access the data *n*-times before *t*. However, if the present time $t^{\prime }$ exceeds *t*, even if the current number of accesses *i* does not exceed *n*, the DU cannot pass the permission verification. The specific process and results of each verification are shown in Table 3. When the access time limit is exceeded, no matter how much the user tries, the verification cannot be passed, and $v_{1}$ and $v_{2}$ will not be updated.

### 5.2. Simulation 2: Scenario Simulation Experiment

**Figure 6:**
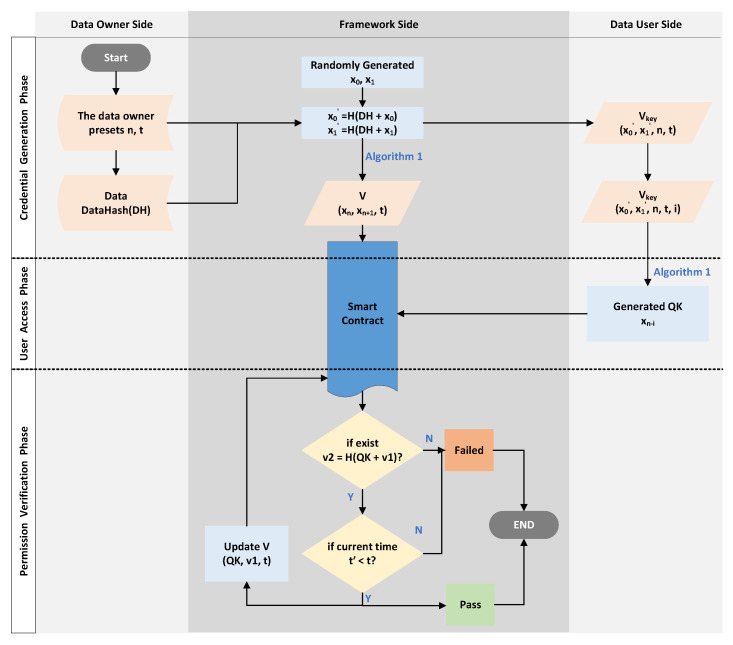
The illustration of Experimental Simulation 2.

This simulation was designed to evaluate the performance of the proposed approach in this paper. The simulation still followed the design of the previous simulation in Section 5.1, but with some changes in the details, as shown in Figure 6:

1. The same as the first step in the simulation in Section 5.1, set the same *n* and *t*.
2. Then, when the framework receives an access request to the data, it generates two random numbers $x_{0}$ and $x_{1}$ and uses Algorithm 1 to calculate $x_{0}^{\prime }=H\left(DH+x_{0}\right)$, where *H* is the same hash function as in Algorithm 1, DH is the hash value of the data; calculate $x_{1}^{\prime }$ similarly. This way prevents the framework from generating the same two sets of random numbers, although this is unlikely. Then, use Algorithm 1 to calculate $x_{n}$ and $x_{n+1}$, and send *V* and $V_{key}$ to the ACC and the requesting DU, respectively.
3. The DU uses $V_{key}$ and Algorithm 1 to calculate QK. Verify whether the DU can successfully pass the data access permission verification and record the time required for the ACC to complete a verification judgment.

**Figure 7:**
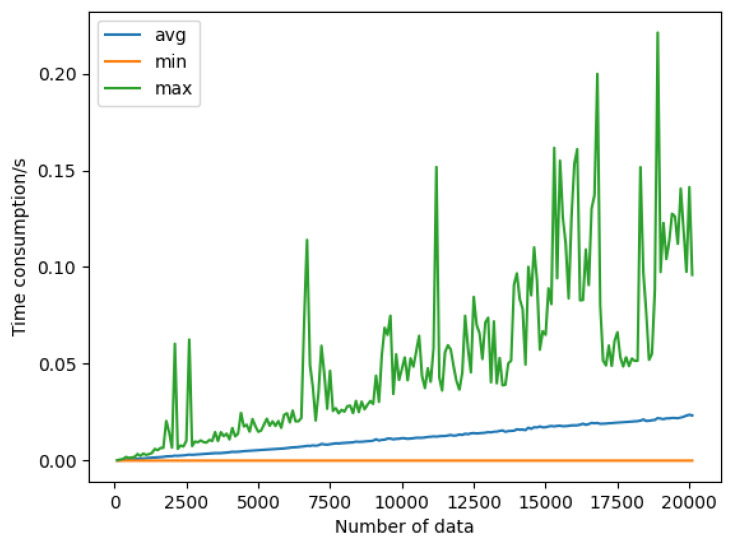
Simulation 2’s results.

Evaluate the performance impact on the verification as the number of data objects grows while the number of *V* stored in the ACC grows as well. The simulation results are shown in Figure 7. The minimum time required for verification was almost unaffected, while the average time required was linearly and positively correlated with the number, and the maximum time required fluctuated wildly. This is because the n-times access control approach in this paper needs to iterate through all *V* in the contract. When *V* is precisely at the first position of the list, it only needs to be calculated once to pass the verification The maximum time required for verification fluctuated wildly and was related to the size of the data themselves. If the amount of data is large, it will take a long time to calculate the hash, and when the amount of data is small, the hash calculation time can be almost ignored. Overall, the average time required for verification increased steadily with the growth of *V*, indicating that the amount of data had a certain impact on the performance of this approach.

It can be presumed that, when the amount of *V* grows to a certain level, the time consumption cost will become unacceptable. Consider deploying multiple ACC contracts and controlling the maximum amount of *V* in each ACC to mitigate the time cost.

### 5.3. Simulation 3: Online Usage Costs’ Simulation Experiment

**Figure 8:**
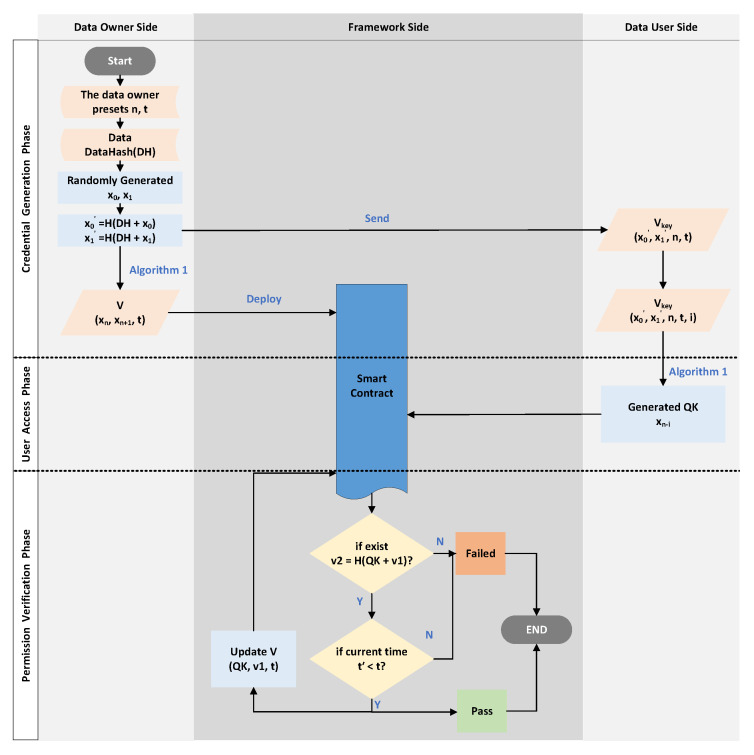
The illustration of Experimental Simulation 3.

Besides using the proposed framework in this paper, the n-times access-control mechanism proposed in this paper can also be implemented in public blockchains and smart contracts. However, it should be noted that, due to the lack of a “voucher-generation module” on the public blockchain, the generation of random numbers $X_{0}$ and $X_{1}$ cannot be realized. Therefore, this requires the data owner to generate it and send it to the user through other ways and use the API provided by the public blockchain to deploy the contract. The specific process is shown in Figure 8.

This simulation mainly verified the gas consumption when the approach proposed in this paper was used on a public blockchain. For that, we built an Ethereum test net locally to simulate gas consumption in the simulation.

The ACC is mainly responsible for verifying the access permissions and returning true and false. It mainly includes two functions, AddVoucher() and AccessVerification(). Since determining access rights requires traversing all stored vouchers *V* in the contract, the value of the gas consumption of the AccessVerification() function increases as the number of vouchers *V* stored in the contract increases. In contrast, the gas consumption of AddVoucher() is almost constant for the same size of data.

**Figure 9:**
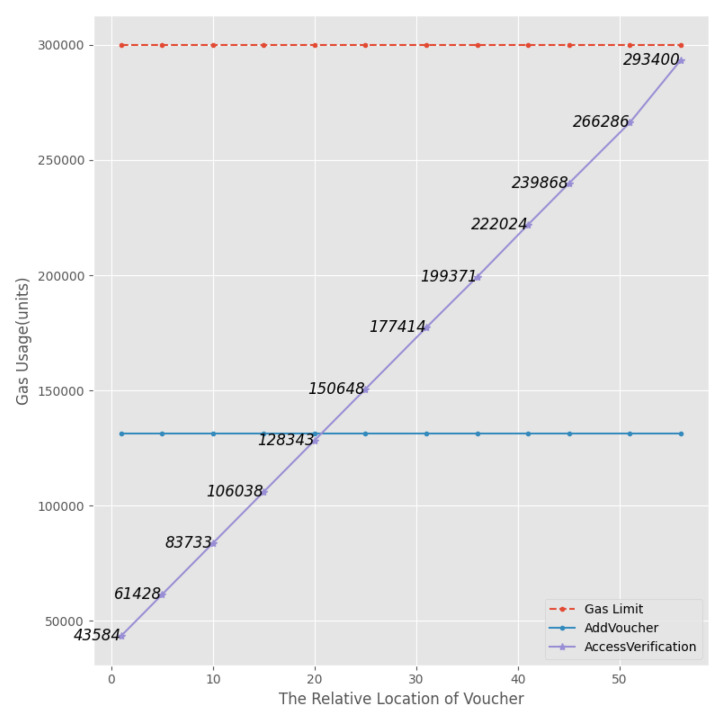
Simulation 3’s results.

The ACC’s gas consumption is shown in Figure 9 (assume the gas limit is 300,000 units). The amount of gas consumption in a single call to AddVoucher() is almost constant at 131,341 units and does not vary with the position of the user vouchers stored in the smart contract. The gas consumption of AccessVerification() increases as the number of vouchers *V* increases, as inferred.

## 6. Conclusions

In this paper, we proposed a blockchain-based access-control framework with configurable restrictions, which can be used for the trusted management of data-driven cyber–physical systems. Meanwhile, we proposed an n-times access-control approach with smart contracts and a hash chain. The framework and approach enable data owners to host their data and achieve the goal of offline one-to-many data access control, and it can limit the number of times a user can access during the deadline. Additionally, our blockchain-based framework also achieves trusted audits and ensures the non-repudiation of records.

However, our approach still has some weaknesses, for instance: (1) Since the storage method of the blockchain is continuous “adding”, there is no “modification” and “deletion”. Then, with continuous use, the stored records will become very redundant, and how to deal with records that are too old is still a problem. (2) Blockchain-based access control systems are limited to the performance issues of the blockchain itself. When the system access is very frequent and highly concurrent, it is very likely that it will not work properly. (3) Access credentials are the only credentials for data users. Once lost, they cannot be retrieved or may be used maliciously by others.

In the following work, we plan to design a secure data marketplace that combines techniques such as proxy re-encryption, differential privacy, and zero-knowledge proofs, which guarantees secure distribution, access control, and privacy protection of data.

## Figures and Tables

**Table 1:** Comparison of access control frameworks.

Access Control Model	Reference	Advantages	Disadvantages
Discretionary Access Control (DAC)	[11,12,13]	Flexible user-based authorization	Insufficient restriction for enforcing information flow policies; lacks scalability
Mandatory Access Control (MAC)	[14,15,16]	Maintains security levels for different objects hierarchically	Central authority for access determination; lacks transparency
Role-Based Access Control (RBAC)	[18,19,20]	Simplified access management based on user roles	Lacks scalability; hard-to-change permission–role assignments
Blockchain-Based Access Control	[9,22,23,29]	Decentralized access control using blockchain; transparency, immutability, and smart contracts are achieved	Challenges with decryption key management; limited applicability due to computational restrictions

**Table 2:** Detailed results of Simulation 1 (Part A).

x0: 256511764204057886305672299344854953792; x1: 66196481555002381006091047960932182450; n: 8; t: 2022-11-01 23:59:59				
**#**	**User**	**Smart Contract**		**Verification**
	**QK**	**v1**	**v2**	
1	217545eb7ccb335ff24f2598fbac590c4775a5610bfecfc55e79b2508a468e17	0108bd640de4c703d9111dbcb80ec05937c7d0f14911ba00e64d5e7a3fe85c00	9503728b9a69ba06f921eb2cd79928112dfb9d402fd3a2629fe6021d7e98cdf8	PASS
2	6b012782426f86568f533457d7127b220606fa638486ff4d400e697c1d770977	217545eb7ccb335ff24f2598fbac590c4775a5610bfecfc55e79b2508a468e17	0108bd640de4c703d9111dbcb80ec05937c7d0f14911ba00e64d5e7a3fe85c00	PASS
3	93cb687dcd960c880c249b0daf29a53b3492b2fbd3cf26c6156cd88fe77785cf	6b012782426f86568f533457d7127b220606fa638486ff4d400e697c1d770977	217545eb7ccb335ff24f2598fbac590c4775a5610bfecfc55e79b2508a468e17	PASS
4	97588264857b88245e731b7e21cab3ba64ef7e96a1783368e29c0a6dc6fead0e	93cb687dcd960c880c249b0daf29a53b3492b2fbd3cf26c6156cd88fe77785cf	6b012782426f86568f533457d7127b220606fa638486ff4d400e697c1d770977	PASS
5	1470c187c88217b152305693779128e9e7da74dab8b1b3e969cd71e08e2884fa	97588264857b88245e731b7e21cab3ba64ef7e96a1783368e29c0a6dc6fead0e	93cb687dcd960c880c249b0daf29a53b3492b2fbd3cf26c6156cd88fe77785cf	PASS
6	7e8e1ed28f7bc36c17174b448b52d036e62d6f77b3ce083c6f03e6e8f025b9e4	1470c187c88217b152305693779128e9e7da74dab8b1b3e969cd71e08e2884fa	97588264857b88245e731b7e21cab3ba64ef7e96a1783368e29c0a6dc6fead0e	PASS
7	66196481555002381006091047960932182450	7e8e1ed28f7bc36c17174b448b52d036e62d6f77b3ce083c6f03e6e8f025b9e4	1470c187c88217b152305693779128e9e7da74dab8b1b3e969cd71e08e2884fa	PASS
8	256511764204057886305672299344854953792	66196481555002381006091047960932182450	7e8e1ed28f7bc36c17174b448b52d036e62d6f77b3ce083c6f03e6e8f025b9e4	PASS
9	233879529224677071018539504275645510501	256511764204057886305672299344854953792	66196481555002381006091047960932182450	Failed
10	199023813661482065363584570395659124763	256511764204057886305672299344854953792	66196481555002381006091047960932182450	Failed
11	76666569808458007876413332894573616585	256511764204057886305672299344854953792	66196481555002381006091047960932182450	Failed
12	28654509403185559621785586756093836683	256511764204057886305672299344854953792	66196481555002381006091047960932182450	Failed
13	150549715489856463120275463270756442252	256511764204057886305672299344854953792	66196481555002381006091047960932182450	Failed
14	193898710617987455574699607267857855265	256511764204057886305672299344854953792	66196481555002381006091047960932182450	Failed
15	313952248899608118652593662097449699480	256511764204057886305672299344854953792	66196481555002381006091047960932182450	Failed

**Table 3:** Detailed results of Simulation 1 (Part B).

x0: 258740906750448359793664013205900417100; x1: 21417340383127709937124895685701875352; n: 8; t: 2022-09-01 23:59:59				
**#**	**User**	**Smart Contract**		**Verification**
	**QK**	**v1**	**v2**	
1	624ee075d2fad0f8f12279ac5a46766456aa57a8eafd6c2cfac6ed785cc89031	54f5276bd0e01ae56795f7c2f3eaed59ace180963d16130fe9bd051d69b7c79f	7551d3a3db30e68770898af077805b8a310d66e69d4c21cc545912cfb22c14c9	Failed
2	bda9c5fad18ea6d9c4996edc56879a063777af4f39a225ae226ad5be73d3aa8e	54f5276bd0e01ae56795f7c2f3eaed59ace180963d16130fe9bd051d69b7c79f	7551d3a3db30e68770898af077805b8a310d66e69d4c21cc545912cfb22c14c9	Failed
3	e597d40b67aebe57cbe65eb394991c5c2cb5ce4316122534ae84c8420a9bbaa3	54f5276bd0e01ae56795f7c2f3eaed59ace180963d16130fe9bd051d69b7c79f	7551d3a3db30e68770898af077805b8a310d66e69d4c21cc545912cfb22c14c9	Failed
4	513640421035397a1ec10927e507063086e2c4a94a441ba08dd0680f50b1a69f	54f5276bd0e01ae56795f7c2f3eaed59ace180963d16130fe9bd051d69b7c79f	7551d3a3db30e68770898af077805b8a310d66e69d4c21cc545912cfb22c14c9	Failed
5	3a94ff6e2e22bc0c8402cb4108ad17fd0962f2d53a01cd2d307d76f666c00d89	54f5276bd0e01ae56795f7c2f3eaed59ace180963d16130fe9bd051d69b7c79f	7551d3a3db30e68770898af077805b8a310d66e69d4c21cc545912cfb22c14c9	Failed
6	649555ab2f0f3634a685d3699ffc7a36063f6f79eeb2ab3a1ceb671e536197ee	54f5276bd0e01ae56795f7c2f3eaed59ace180963d16130fe9bd051d69b7c79f	7551d3a3db30e68770898af077805b8a310d66e69d4c21cc545912cfb22c14c9	Failed
7	21417340383127709937124895685701875352	54f5276bd0e01ae56795f7c2f3eaed59ace180963d16130fe9bd051d69b7c79f	7551d3a3db30e68770898af077805b8a310d66e69d4c21cc545912cfb22c14c9	Failed
8	258740906750448359793664013205900417100	54f5276bd0e01ae56795f7c2f3eaed59ace180963d16130fe9bd051d69b7c79f	7551d3a3db30e68770898af077805b8a310d66e69d4c21cc545912cfb22c14c9	Failed
9	333054958953516216718308573203038522709	54f5276bd0e01ae56795f7c2f3eaed59ace180963d16130fe9bd051d69b7c79f	7551d3a3db30e68770898af077805b8a310d66e69d4c21cc545912cfb22c14c9	Failed
10	75481318761718134747663483379473610349	54f5276bd0e01ae56795f7c2f3eaed59ace180963d16130fe9bd051d69b7c79f	7551d3a3db30e68770898af077805b8a310d66e69d4c21cc545912cfb22c14c9	Failed
11	231127855926238800774132249040644381881	54f5276bd0e01ae56795f7c2f3eaed59ace180963d16130fe9bd051d69b7c79f	7551d3a3db30e68770898af077805b8a310d66e69d4c21cc545912cfb22c14c9	Failed
12	231726135973836923017065638586780767802	54f5276bd0e01ae56795f7c2f3eaed59ace180963d16130fe9bd051d69b7c79f	7551d3a3db30e68770898af077805b8a310d66e69d4c21cc545912cfb22c14c9	Failed
13	271609142731823061182786813952755759617	54f5276bd0e01ae56795f7c2f3eaed59ace180963d16130fe9bd051d69b7c79f	7551d3a3db30e68770898af077805b8a310d66e69d4c21cc545912cfb22c14c9	Failed
14	230894908861042681808866636663680642987	54f5276bd0e01ae56795f7c2f3eaed59ace180963d16130fe9bd051d69b7c79f	7551d3a3db30e68770898af077805b8a310d66e69d4c21cc545912cfb22c14c9	Failed
15	48569152719937234965684312602145656412	54f5276bd0e01ae56795f7c2f3eaed59ace180963d16130fe9bd051d69b7c79f	7551d3a3db30e68770898af077805b8a310d66e69d4c21cc545912cfb22c14c9	Failed
